# 

**Published:** 1887-03-12

**Authors:** 


### The Hospital.

An Institution, Family, and Parochial Journal of Hospitals, Asylums, and all Agencies for the Care of the Sick, Criticism and News.

SATURDAY, MARCH 12TH, 1887.

### The Literary Prize.

A PRIZE of **Two Guineas** will be given every month for the best story or incident based upon hospital, or asylum, or student life, or any incident connected with the professions of medicine or nursing.

### Hospital Housekeeping.

Forms and full particulars of the Quilter Prizes under this head (see page 355 for 19th Feb.) may be had on application to the Editor of The Hospital, Norfolk House, W.C. All competitive essays to be sent in by 30th April, 1887. Prizes, £10 10*s*. and £5 5*s*.

The Children's Corner and Amusements with Profit will be found on page vi of this week's issue.

**Notice to Correspondents.**

BLUE STOCKING is reminded that correspondents must send their names and addresses, not necessarily for publication, but as a pledge of good faith.

### The In- and Out-Patients' Hospital Diary.

This Diary is so arranged as to make some portion of it appear every week, and the whole in each monthly part. It has been very difficult to compile, and corrections will at all times be gratefully received. It has been suggested that similar information about the larger Provincial and Scotch Hospitals would be useful to many readers. We should be glad to hear if this is felt to be the case.

### GENERAL HOSPITALS.—*continued.*

| Hospitals and Terms of Admission. | Physicians, Surgeons, and Medical Officers, with Days and Hours of Attendance. |
|---|---|
| St. Mary's Hospital, Cambridge Place, Paddington, W. Sec., Mr. Pietro Michelli<br>In-patients are admitted by letter of recommendation. Urgent cases and accidents free at all times. Out-patients require a letter. Electrical treatment Tu. and F. 2 | *In-Physicians*, Sir Edward Sieveking, Tu. F. 1.45; Drs. Broadbent, M. Th. 1.45; Cheadle, W. 1.45, S. 9.30<br>*In-Surgeons*, Messrs. Norton, M. Th. 1.45; Owen, Tu. F. 1.45; H. Page, W. S. 1.45<br>*Out-Physicians*, Drs. D. B. Lees, W. S.; S. Phillips, M. Th; R. Macguire, Tu. F. Hour, 1.30<br>*Out-Surgeons*, Messrs. W. Pye, M. Th.; A. J. Pepper, Tu. F.; A. Q. Silcock, W. S. Hour, 1.30 |
| St. Thomas's Hospital, Westminster Bridge Road, S.E. Steward, Mr. F. Walker<br>Accidents and emergencies free at all times. Other cases by Governor's letter, but, where unobtainable, application should be made to the Steward by letter, or personally between 10 and 4. Vaccination on W. at 11.30 | *In-Physicians*, Drs. Bristowe, Tu. F.; Stone, M. Th.; Ord, M. Th.; J. Harley, Tu. F.; Gervis, Tu. F. Hour, 2<br>*In-Surgeons*, Messrs. Sydney Jones, Tu. F.; Croft, M. Th.; Sir W. MacCormac, M. Th. Hour, 2<br>*Out-Physicians*, Drs. Payne, Tu. F.; Sharkey, M. Th.; Gulliver, W. S. Hour, 1.30<br>*Out-Surgeons*, Messrs. Clutton, Tu. F.; Anderson, W. S.; Pitts, M. Tu. Hour, 1.30 |
| Tottenham Training Hospital, Tottenham, N. Director, Dr. Laseron<br>Free. Accidents and emergencies admitted at all times | *In-Physician*, Dr. Rasch, Tu. F. 3 to 5<br>*In-Surgeons*, Drs. Lichtenberg, M. Th. 3 to 5; E. H. May, M. Th. 3 to 5<br>*Out-Surgeon*, Dr. Lloyd Smith, M. W. 11 to 2; *Men* only, W. 7 to 8 p.m. |
| University College Hospital (or North London), Gower Street, W.C. Sec., Mr. N. H. Nixon<br>Accidents and emergencies free. All other cases by Governor's letter of recommendation | *In-Physicians*, Drs. W. Fox, Tu. Th. 2, S. 9.30; Ringer, M. W. F. 1.30; Bastian, M. 2.30, W. 10, F. 2.30; F. Roberts, Tu. 1.15, Th. 1.30, S. 9.30<br>*In-Surgeons*, Messrs. B. Hill, Tu. 2.15, W. 2, F. 2.15; C. Heath, M. W. F. 2; M. Beck, Tu. Th. S. 2<br>*Out-Physicians*, Drs. Gowers, W. S.; Poore, Tu. F.; Barlow, M. Th. Hour, 1.30<br>*Out-Surgeons*, Messrs. Barker, M. Th.; Godlee, Tu. F.; Horsley, W. S. Hour, 1.30 |
| West London Hospital, Hammersmith Road, W. Sec. and Supt., Mr. R. J. Gilbert<br>Accidents and emergencies free at all times. Other cases require a letter of recommendation. New cases must attend at 1.30 | *In-Physicians*, Drs. G. Rogers, D. W. Hood, F. Drewitt<br>*In-Surgeons*, Messrs. C. Keetley, F. Edwards, W. B. Clarke<br>*Out-Physicians*, Drs. Herringham, M. Th.; Ball, Tu. F.; S. Taylor, W. S. Hour, 2<br>*Out-Surgeons*, Messrs. Ballance, Tu. F.; Weiss, M. Th.; Wainewright, W. S. Hour, 2 |
| Westminster Hospital, Broad Sanctuary, S.W. Sec., Mr. S. M. Quennell<br>Accidents and urgent cases free at all times. Other cases require a letter of recommendation | *In-Physicians*, Drs. Sturges, M. Th. S.; Allchin, M. Tu. F.; Donkin, Tu. W. S. Hour, 1.30<br>*In-Surgeons*, Messrs. Cowell, M. Tu. Th.; Davy, M. Tu. F.; Macnamara, M. W. S. Hour, 1.30<br>*Out-Physicians*, Drs. De H. Hall, M. Th.; A. H. Bennett, Tu. F.; Murrell, W. S. Hour, 1.30<br>*Out-Surgeons*, Messrs. Cooke, M. Th.; Bond, Tu. F.; Barrow, W. S. Hour, 1.30 |

### II.—SPECIAL HOSPITALS.

### CANCER.

| | |
|---|---|
| Ancer Hospital, Brompton, S.W. Sec., Mr. W. H. Hughes<br>Entirely free to both in- and out-patients | *In- and Out-Surgeons*, Drs. A. Marsden, W.; H. Snow, M.; F. A. Purcell, F.; Messrs. F. B. Jessett, W.; C. Stonham, Th.; C. Jennings, Tu.; W. H. Elam, S. Hour, 2 |
| London Hospital, Whitechapel Road, E. | Daily, 1.30. *See* General Hospitals |
| Middlesex Hospital, Berners Street, W. Admission free | *In-Surgeons*, Messrs. Hulke, Lawson, and Morris<br>*Out-Surgeon*, Mr. Morris, Th. 1.30 |
| St. Saviour's Hospital, Osnaburg Street, N.W. Sec., Mr. E. Poitiers<br>By Governors' letters | *In- and Out-Physician*, Dr. A. Kennedy, 10.30 and 4 daily |

### CHILDREN'S DISEASES.

| Hospitals and Terms of Admission. | Physicians, Surgeons, and Medical Officers, with Days and Hours of Attendance. |
|---|---|
| Alexandra Hospital for Children, 18, Queen Sq., W.C. Sec., Mrs. Marsh<br>For hip disease | *Physician*, Dr. Steavenson<br>*Surgeons*, Messrs. H. Marsh Tu. F.; A. Bowlby, M. Th. Hour, 4.30<br>(No out-patients) |
| All Saints' Childrens Hospital, 4, Margaret Street, W.<br>For chronic joint diseases | *Surgeon*, Mr. N. Smith<br>(No out-patients) |
| BelgraveHosp.forChildren, 77 & 79, Gloucester Street, Pimlico. Hon. Secs., Capt. Stopford, and Rev. J. Storrs<br>By letters; accidents free | *In- and Out-Physicians*, Drs. W. Hope and W. Ewart, M. Th. 9<br>*In- and Out-Surgeons*, Messrs. C. T. Dent and Margerison, Tu. F. 9 |
| Charing Cross Hospital, West Strand, W.C. | *Out-Physician*, Dr. Nursby, W. S. 1.30 |
| Cheyne Hospital for Sick and Incurable Children, 46, Cheyne Walk, Chelsea, S.W. Sec., Miss D. Evans | *Physician*, Dr. G. V. Poore<br>*Surgeons*, Messrs. T. P. Bartlett and J. Macready attend alternate days in afternoons. (No out-patients) |
| East London Hospital for Children and Dispensary for Women, Glamis Road, Shadwell, E. Sec., Mr. Ashton Warner<br>By Governor's letter. Urgent cases and accidents are admitted free | *In-Physicians*, Drs. Donkin, M. Th.; Eustace Smith, T. F.; Crocker, F.<br>*In-Surgeons*, Messrs. Calder, W. F.; Parker, W. F.<br>*Out-Physicians*, Drs. Crocker, M.; Williams, Tu. Th.; Coutts, W. F. Hours, 1 and 3<br>*Out-Surgeon*, Mr. Dunn, Tu. F. 1, 3 |
| Evelina Hospital for Sick Children, Southwark Bridge Road, S.E. Sec., Mr. T. S. Chapman.<br>Free, but Governor's letter takes precedence. In-patients (boys) between 2 and 10, (girls) between 2 and 12 | *In-Physicians*, Drs. F. Taylor and J. Goodhart<br>*In-Surgeons*, Messrs. H. G. Howse and R. C. Lucas<br>*Out-Physicians*, Drs. N. Tirard, M. Th. 9; F. Willcocks, Tu. F. 9<br>*Out-Surgeons*, Messrs. C. J. Symonds, S. 9; G. H. Makins, W. 9 |
| German Hospital, Dalston Lane, Dalston, E. | *In-Physician*, Dr. Rasch, M. Th. 9 |
| Great Northern Central Hospital, Caledonian Rd. N. | *Physicians*, Drs. Murray and Barnes, W. 2.30 |
| Grosvenor Hospital for Women and Children, Vincent Sq., S.W. Hon. Secs., Mr. A. J. Ram and Miss Day<br>By letter or payment | *In- and Out-Physicians*, Drs. R. Gibbons and Steavenson, Tu. W. Th. S. 2<br>*In- and Out-Surgeons*, Messrs. Smythe and Parker, Tu. W. Th. S. 2 |
| Hospital for Sick Children, 48 and 49, Great Ormond St., W.C. Sec., Mr. S. Whitford<br>By Governors' letters. If without letter, the case of the applicant is investigated<br>In-patients between 2 and 12; Out-patients under 12 | *In-Physicians*, Drs. W. Cheadle, Tu. F.; Sturges, W. S.; Barlow, M. Th. Hours, 9 to 11<br>*In-Surgeons*, Messrs. H. Maish, W. S.; E. Owen, W. S. Hours, 9 to 11<br>*Out-Physicians*, Drs. R. J. Lee and Abercrombie, M. Th.; Lubbock and Money, Tu. F. Hours, 8.30 to 10<br>*Out-Surgeons*, Messrs. Morgan and Pitts, W. S. 8.30 to 10 |
| King's College Hospital, Portugal Street, W.C. | *In- and Out-Physician*, Dr. T. C. Hayes, W. F. 1 |
| Middlesex Hospital, Berners Street, W.<br>Free. Children under 3 | *Out-Physician*, Dr. Duncan, W. S. 1.30 |
| North Eastern Hospital for Children, Hackney Road, E. Sec., Mr. A. Nixon<br>By free ticket or small payment | *In- and Out-Physicians*, Drs. W. Cayley, F. Turner, C. Semple, and W. Pasteur, M. W. Th. S. 1.30<br>*In- and Out-Surgeons*, Messrs. Waren Tay and Pollard, M. 1.30, F. 9 |
| Paddington Green Children's Hosp., Paddington Green. Sec., Mr. W. H. Pearce<br>Admission free; boys under 12; girls under 14 | *In- and Out-Physicians*, Drs. T. Lauder Brunton, Ogilvie, Tu. F.; G. Laycock, M. Th.; S. Phillips, W. S. Hour, 9.15<br>*In- and Out-Surgeons*, Messrs. W. W. Cheyne, M., S. Boyd, F. Hour, 9.15 |
| Royal Hospital for Children and Women, Waterloo Bridge Road, S.E. Sec., Mr. R. G. Kestin<br>Out-Patients pay 1*d.* on each attendance. Boys not admissible, as out-patients, over 12, or, as in-patients, over 8 | *In-Physicians*, Drs. Park, M. Th.; Gulliver, Tu. F.; Price, W. S. Hour, 2<br>*In-Surgeon*, Mr. W. H. A. Jacobson, M. Th. Hour, 2<br>*Out-Physicians*, Drs. Park, M. Th.; Dakin, W. F.; Hadden, Tu. S. Hour, 2<br>*Out-Surgeon*, Mr. E. O. Day, W. 2 |
| St. Mary's Hospital, Cambridge Place, Paddington | *Out-Physician*, Dr. M. Handfield Jones, M. Th. 1.30 |
| St. Thomas's Hospital, Westminster Bridge Road, S.E | *Out-Physician*, Dr. Cory, W. S. 1.30 |

### THE CHILDREN'S CORNER.

### OUR PASTIMES.

### Eleventh Competition.

No. 57. PUZZLE SENTENCE. The following sentence will give you the name of a popular author; "Chickens are *£ s. d.*" Who is he?

No. 58. CONUNDRUM. What is that which gives a cold, cures a cold, and pays the doctor?

* * * *
* * * * No. 59. SQUARE WORD. A precious metal; a
* * * * German river; a Siberian river; a vehicle.
* * * *

No. 60. ENIGMA.

Three feet I have,
But ne'er attempt to go,
And many nails thereon,
But not one toe.

No. 61. FIGURE WORD. Convert the following into a word, $5+1+10+\frac{2}{3}$ of 10.

### Results of Ninth Competition.

No. 47. CONUNDRUM. The difference between your Puzzle Editor and one of the Unemployed is very simple. The ormer lives to see how he can puzzle, while the latter is puzzled to see how he can live.

Some other very good answers, for which I give credit, have reached me. Skye suggests, "The one gives work, and the, other seeks it;" Peeler: "The one finds employment in puzzles, the other is puzzled to find employment;" Alsie: "The unemployed want work, the Puzzle Editor wants workers;" Scrooge: "One gives employment and the other seeks it;" Tim: "One works his brain to get puzzles, and the other puzzles his brain to get work;" and Mikado: "One lives by his wit, the other wits not how to live."

No. 48. DOUBLE MESOSTICH.

M
c A t
d a T e s
M A T C H E S
e t H e r
t E a
S

No. 49. AN ARITHMETICAL QUESTION. At a first glance, it seems that half-a-dozen dozen and six dozen dozen should be the same, but a little reflection shows that the former is equivalent to only 72, while the latter is 864; the difference is therefore 792.

No. 50. DECAPITATION. The words here are That—hat; Fire—ire; Cloth—loth.

No. 51. BURIED PROVERB. "Make hay while the sun shines."

*All right*—Florrie, Skye, Peeler, Alsie, Ellie, Scrooge, A. C. K., Tim, P.S., Mikado; *Four right*—Isobel, Paul Methven, Joe, Sutton, Dot; *Three right*—Ariadne, Little Mary, Bismarck.—Isobel is credited with five right answers, in letter wrongly addressed last week.

### Letters from Little Folk.

The subject for next week's letters will be "School Studies." You may tell me something about what you learn, your favourite subjects, and so on. The letters about "Friends" were exceedingly good. Here are two or three.

DEAR MR. EDITOR.—"A friend in need is a friend indeed." How true that saying is, and how one comes to know it all through life when one has got a true friend. At school the friendship begins, first in small matters, such as lessons, and difficulties of school life. Then at the university, friends work together; take their degree together, and so on all through life. Tom Brown is a good example of a friend; how he defended little Arthur all through his school life, and any one who has read the book will remember how he was repaid.

Eaton, Chester. Yours truly, PEELER, aged 17.

DEAR MR. EDITOR,—I suppose you think we all have one or two *friends* whom we like better than the rest, and that we can tell you a lot about them and their ways; but, though I have plenty of acquaintances, and like all my schoolfellows, I have not got any intimate *friend* among them; but I have a brother, a little older than myself. We do the same lessons, read each other's books, and, on the whole, are very good *friends.* I cannot get him to join in the competition, for he says he does not like guessing puzzles, and cannot bear writing letters.—Believe me, your little *friend*, TIM.

25, Dornton Road, Balham.

(Please append age in next letter.)

DEAR MR. EDITOR,—I have a great many friends, most of them younger than myself, and nearly all of them live close to me, and go to the same school, which is very nice, because we can walk together, and talk about studies, or any little secrets that we may have to tell. The chief of my *little* friends are two little French girls, Marguerite and Louise; the one is six, and the other four years old. They are dear little things, and so obedient. It is so amusing to hear them talk French to each other.—I am, yours very sincerely,

20, St. John's Villas, East Dulwich. FLORRIE, aged 9.

### Letter Prize.

One mark each: Florrie, Skye, Peeler, Ellie, Scrooge, Tim, Mikado, P.S., Halford, Isobel.

### Notices to Correspondents.

SCROOGE.—I am so sorry to hear you have such a bad cold. I much hope you will soon be well again.

MIKADO.—Your young friends are very musical. I should think the one who plays the flute so beautifully must be exceedingly clever.

SKYE.—Your letter about your brothers and sister much interested me. What a misfortune that was to Alice's poor dolly!

Answers must be addressed to the "PUZZLE EDITOR," THE HOSPITAL, Norfolk House, Norfolk Street, Strand, W.C., not later than Thursday next. The "Children's Corner" Coupon (see advertisement pages) must be enclosed.

The address and full Christian name and surname, and the age of the competitor, must be stated in all replies. A *nom de plume* may be added for the purposes of publication.

### AMUSEMENTS WITH PROFIT.

### PRIZE COMPETITIONS.

### Quarterly Prize Competition.

Began 22nd January; Ends 16th April.

**A Prize of Three Guineas**

will be awarded to the Competitor who shall have sent in the largest number of correct or best answers. No one will be permitted to win the Three Guinea Prize twice in any one year, but we shall award annually an additional

**Prize of Five Guineas**

to the competitor who shall have sent in the largest number of correct or best answers during the twelve months.

### No. 15. Double Acrostic.

The recent earthquake this has wrought;
In far New Munster I am sought;
A coin; a ruler we obey;

A City; a hero of renown;
In Moravia, an ancient town;
The proper thing, Blue Ribbonists say;

A brewer, I think we all well know;
This line I'm sure the light will show;
A famous singer of the day;

A tortuous stream from Iseo flows;
This last the poor Riviera shows.

(The eleven initials and finals give the name of an institution at Brompton devoted to what has been truly named our "national disease").

### No. 16. Mathematical Question.

A man buys larks at 3*d.* each, wrens at $\frac{1}{2}$*d.* each, and sparrows at $\frac{1}{4}$*d.* each. He lays out 1*s.* 8*d.* in all. How many of each kind of bird did he buy?

### Answers.

No. 11. DOUBLE ACROSTIC.

| | | |
|---|---|---|
| N | angasak | I |
| E | de | N |
| W | ol | F |
| C | apr | I |
| A | xminste | R |
| S | ia | M |
| T | e | A |
| L | ea | R |
| E | l | Y |

Correct answers have been received from Perseverance, Mr Bedingfeld, H. J. C., Miss Larkins, Mr. J. High, Tabs, Ostrich Mater, Patient, Hexameter, A. M. E., Peeler, The E. D. A. K. M., Ellice Gretta, Robe, Paddy, Doris, Gip, Condorcet.

No. 12. NURSE.

Again most of our competitors have chosen verse in which to express their definition of "Nurse." It is surprising how very few of the replies described the nurse as the doctor's handmaiden.

Best answers have been received from Gip, Condorcet Paddy, Robe, Gretta, K. M., Ellice, The E. D. A., Peeler Patient, A. M. E., Ostrich, Mater, Tabs, Frederick C., H. J. C Mrs. Bedingfeld, Perseverance.

### Notices to Correspondents.

THE E. D. A.—Your full name and address must be enclosed next time.

PADDY.—The two names you mention cannot certainly be classed as those of known composers.

A. M. E.—The half clearly occurs thrice ($147\frac{1}{2}+\frac{1}{2}$, $73\frac{1}{2}+\frac{1}{2}$, $36\frac{1}{2}+\frac{1}{2}$). Your solution is evidently wrong, since your sales, plus 36, do not make 281 but 284.

Answers must be addressed to THE PRIZE EDITOR, AMUSEMENTS WITH PROFIT, THE HOSPITAL, Norfolk House, Norfolk Street, Strand, W.C., not later than Saturday next. The full name and address must in all cases be given, but a *nom de plume* may be added for publication. Only one side of the paper should be written on. The "Amusements with Profit" Coupon (see advertisement pages) must be enclosed with replies.

